# Color Stability and Surface Roughness of Two Restorative Material–Production Systems: Effects of Surface Finishing and Brushing with Two Commercial Toothpastes with Different RDA Classifications

**DOI:** 10.3390/dj14100674

**Published:** 2026-10-16

**Authors:** Nevra Nur Çiftçi, Özgül Karacaer

**Affiliations:** Department of Prosthodontics, Faculty of Dentistry, Gazi University, Ankara 06490, Türkiye; ozgulkaracaer1@yahoo.com

**Keywords:** color stability, CIEDE2000, surface roughness, additive manufacturing, subtractive manufacturing, resin-based restorative materials, simulated toothbrushing, surface finishing

## Abstract

**Background/Objectives:** This in vitro study compared color and roughness changes after simulated toothbrushing in milled GC Cerasmart 270 (CS) and 3D-printed Formlabs Permanent Crown (FL) following glazing or mechanical polishing. **Methods:** Eighty specimens were assigned to eight groups (2 × 2 × 2 factorial design; *n* = 10) using Colgate Total or Colgate Whitening (Colgate-Palmolive Co., Guangzhou, China). Published relative dentin abrasivity (RDA) values were 70 and 124, respectively; RDA was not measured in this study. Color coordinates and mean surface roughness (Ra) were measured before and after 10,000 brushing cycles. CIEDE2000 color differences (Δ*E*_00_) and Ra were analyzed using three-way factorial analysis of variance (ANOVA) and mixed repeated-measures ANOVA, respectively. **Results:** The material–production system × finishing interaction was significant for Δ*E*_00_ (*p* = 0.035; partial η^2^ = 0.060). Mechanically polished FL showed greater color change than mechanically polished CS (mean difference: 0.56; Tukey-adjusted 95% confidence interval (CI): 0.13–0.99). Ra increased after brushing (*p* < 0.001; partial η^2^ = 0.846), with a material–production system × finishing × time interaction (*p* = 0.002; partial η^2^ = 0.127). Among mechanically polished specimens, FL showed a greater Ra increase than CS (mean difference in change: 0.025 µm; Tukey-adjusted 95% CI: 0.006–0.045 µm). No significant toothpaste main effect was detected for either outcome. **Conclusions:** Mechanically polished FL showed greater color change and Ra increase than mechanically polished CS. Mean color differences remained below the acceptability threshold, although some individual specimens exceeded it. Each manufacturing approach and toothpaste condition was represented by one commercial product, preventing separation of manufacturing-method and RDA effects from formulation.

## 1. Introduction

Resin-based definitive restorations can be fabricated by subtractive milling of industrially polymerized blocks or by additive layer-by-layer photopolymerization [1,2,3]. Their mechanical and optical properties depend on both the material formulation and the associated production workflow [1,2,4]. Milled blocks generally have a standardized structure, whereas the properties of materials fabricated by three-dimensional (3D) printing may be influenced by the printer system, layer thickness, build orientation, washing, and post-polymerization procedures [2,4,5,6]. Accordingly, differences between milled and 3D-printed materials should be interpreted within the context of the specific material–production systems evaluated [5]. GC Cerasmart 270 (CS) contains a dimethacrylate matrix reinforced with silica and barium glass, whereas Formlabs Permanent Crown (FL) contains a photopolymerizable dimethacrylate matrix with silanized dental glass (Supplementary Table S1). Matrix chemistry, filler characteristics, and matrix–filler bonding may influence water uptake, degradation, and wear [1]. The refractive-index relationship between matrix and fillers influences translucency and color matching [7]. Post-polymerization conditions can also affect color and microhardness [6]. Extrinsic factors, including surface finishing and brushing with different dentifrice formulations, may further modify roughness, gloss, and color [8,9,10].

Color stability and surface smoothness are important for the clinical performance of esthetic restorations. Color change may compromise esthetics, while increased surface roughness may contribute to gloss loss, staining, and wear. Previous studies have reported material-dependent variations in color stability, surface roughness, microhardness, and wear across 3D-printed definitive resins and computer-aided design/computer-aided manufacturing (CAD/CAM) restorative materials [8,11,12,13]. Mechanical polishing smooths the resin–filler substrate, whereas light-polymerized glaze fills surface irregularities with a separate coating [14]. Brushing with dentifrices may modify surface roughness through matrix wear and filler exposure on polished surfaces or partial coating loss on glazed surfaces [1,15]. Changes in surface texture may influence light reflection [14]; previous studies have also reported color changes associated with surface finishing [16] and toothbrushing [10].

Relative dentin abrasivity (RDA) quantifies abrasivity against dentin and may not directly predict the response of resin-based restorative materials. Abrasive particle characteristics and other formulation components may also influence their surface and optical behavior [10,17,18,19]. Two commercial toothpastes with different reported RDA values were therefore selected to compare products representing different abrasivity conditions [20], without isolating RDA from formulation effects.

Evidence on the combined effects of material–production system, surface finishing protocol, and toothpaste type on color change and surface roughness in definitive resin-based restorations remains limited [6,8,9,10,11,12,13,17,18,19]. Accordingly, this in vitro study aimed to compare the color change (Δ*E*_00_) and mean surface roughness (Ra) of the milled GC Cerasmart 270 and 3D-printed Formlabs Permanent Crown material–production systems following mechanical polishing or glazing protocols and 10,000 cycles of simulated toothbrushing with two commercial toothpastes. The null hypotheses were that material–production system, surface finishing protocol, toothpaste type (two commercial products selected to represent different reported RDA values), and their interactions would have no significant effects on Δ*E*_00_ or Ra.

## 2. Materials and Methods

In this in vitro study, color change and surface roughness after simulated toothbrushing were evaluated for the subtractively manufactured CS and additively manufactured FL material–production systems. Specimens from both systems were subjected to either glazing or mechanical polishing and were subsequently brushed with one of two commercial toothpastes selected to represent different reported RDA values.

CIELAB color coordinates and mean surface roughness (Ra) were measured before simulated toothbrushing and after 10,000 brushing cycles. Color differences between baseline and post-brushing measurements were calculated using the CIEDE2000 color-difference formula (Δ*E*_00_).

GC Cerasmart 270 resin nanoceramic CAD/CAM blocks (GC Corporation, Tokyo, Japan) were used for the subtractive material–production system, whereas Permanent Crown ceramic-filled resin (Formlabs, Somerville, MA, USA), developed for the fabrication of definitive restorations, was used for the additive material–production system. Detailed information on the materials and products used in this study, including manufacturers, lot numbers, compositions, filler/abrasive contents, and relevant processing recommendations, is provided in Supplementary Table S1.

### 2.1. Specimen Preparation

A total of 80 rectangular specimens were prepared from two resin-based restorative materials, with 40 specimens assigned to each material–production system. B1-LT GC Cerasmart blocks were used for the subtractive group, whereas B1 Permanent Crown resin was used for the additive group.

The sample size of *n* = 10 per experimental group was selected based on previous comparable in vitro studies [14,21,22]. No a priori sample-size calculation based on a prespecified primary outcome and clinically relevant effect was performed. The statistical sensitivity of the selected sample size was further assessed using G*Power version 3.1.9.4 (Heinrich Heine University, Düsseldorf, Germany). For a fixed-effects factorial ANOVA comprising eight groups, with α = 0.05 and 80% statistical power, the total sample size of 80 specimens was sufficient to detect effects of at least Cohen’s *f* = 0.317 for 1-degree-of-freedom main effects and interactions. This effect size corresponds to an approximate partial η^2^ of 0.092.

### 2.2. Experimental Group Allocation

The 80 specimens were allocated to eight groups according to a 2 × 2 × 2 factorial design based on material–production system, surface finishing protocol and toothpaste type.

**Figure 1:**
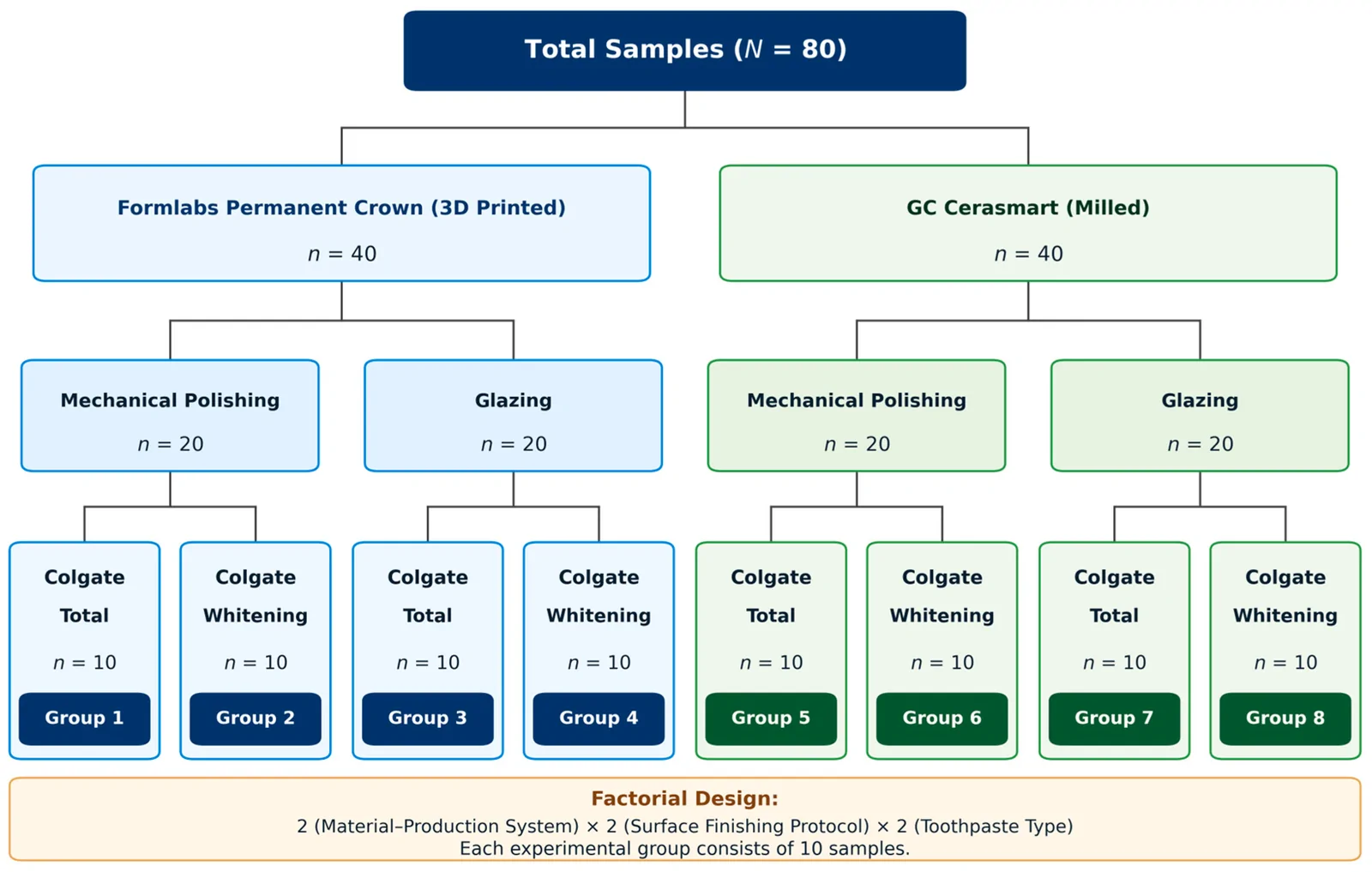
Experimental design and group allocation. Eight groups (*n* = 10 each; *N* = 80) were formed by combining two material–production systems (Formlabs Permanent Crown and GC Cerasmart 270), two surface finishing protocols (mechanical polishing and glazing), and two commercial toothpastes (Colgate Total and Colgate Whitening).

Within each material–production system, 20 of the 40 specimens were mechanically polished and the remaining 20 were glazed. Each finishing group was subsequently divided into two subgroups according to toothpaste type (Colgate Total or Colgate Whitening), resulting in 10 specimens per experimental group. The experimental design and final group coding are presented in Figure 1.

All experimental procedures were performed by a single operator. Within each material–production system, specimens were coded and randomly assigned by drawing lots to the four surface finishing–toothpaste combinations (*n* = 10 per group), without allocation restrictions or blocking according to fabrication block or batch. Specimen positions in the brushing simulator were also randomized. Baseline and post-brushing color and surface roughness measurements were performed on coded specimens, with group allocation concealed from the operator during outcome assessment.

#### 2.2.1. Preparation of the Subtractive Manufacturing Group

GC Cerasmart blocks were sectioned under continuous water cooling using a precision cutting device (Micracut 201, Metkon, Bursa, Türkiye) to obtain specimens with an initial thickness of 1.60 ± 0.05 mm. Specimen thickness was verified using a digital caliper. Forty specimens were obtained from five GC Cerasmart 270 blocks.

The specimens were sequentially ground under water cooling with 600-, 800-, 1000-, and 1200-grit silicon carbide abrasive papers. Each abrasive paper was applied for 10 s using a grinding-polishing device (Gripo 2V Grinder-Polisher, Metkon, Bursa, Türkiye) operating at 200 rpm.

After grinding, the specimens were ultrasonically cleaned in distilled water for 15 min and dried. The subtractive manufacturing workflow is shown in Supplementary Figure S1.

#### 2.2.2. Preparation of the Additive Manufacturing Group

Forty B1-shade specimens measuring 14 × 12 × 1.50 mm were fabricated from Formlabs Permanent Crown resin using a Form 3B+ 3D printer (Formlabs, Somerville, MA, USA). All 40 specimens were fabricated from a single resin lot in one print run on the same build platform and were automatically arranged by the PreForm software (Formlabs, Somerville, MA, USA; https://formlabs.com/software/preform/, accessed on 5 September 2026) with equal spacing. The specimens were positioned vertically, with their broad surfaces oriented at 90° to both the build platform and the free surface of the resin in the tank. Printing was performed using Low Force Stereolithography (LFS) technology at a layer thickness of 50 µm [6,23,24].

After printing, in accordance with the manufacturer’s instructions, the specimens were washed while still attached to their support structures in freshly prepared 98% isopropyl alcohol for 5 min using a Form Wash unit (Formlabs, Somerville, MA, USA). The specimens were then post-polymerized in a Form Cure unit (Formlabs, Somerville, MA, USA) at 60 °C for 20 min. After the initial post-polymerization, the support structures were removed using side cutters, and the specimens underwent a second post-polymerization cycle at 60 °C for 20 min [25]. Following completion of the post-polymerization procedures, the specimens were prepared for the subsequent surface finishing protocols. The additive manufacturing workflow is shown in Supplementary Figure S2.

### 2.3. Surface Finishing Protocols

Specimens from both material–production systems were subjected to either glazing or mechanical polishing. Only one predetermined surface of each specimen was designated as the experimental surface. OPTIGLAZE Color Clear (GC Corporation, Tokyo, Japan) was used for glazing, whereas the two-step Sof-Lex Diamond Finishing and Polishing System (3M ESPE, St. Paul, MN, USA) was used for mechanical polishing, following the manufacturers’ instructions.

#### 2.3.1. Glazing

Residual support remnants on the experimental surfaces of the 3D-printed specimens were removed using a composite polishing rubber. The experimental surfaces of both materials were then airborne-particle abraded with 50 µm aluminum oxide particles for 5 s at 1.5 bar pressure and a 10 mm working distance using an airborne-particle abrasion unit (Heraeus Combilabor CL-FSG 3, Kulzer, Hanau, Germany) [26].

After abrasion, the specimens were ultrasonically cleaned in water for 10 min and dried. G-Multi Primer (GC Corporation, Tokyo, Japan) was applied and dried with clean, oil-free air. OPTIGLAZE Color Clear was then applied as a thin layer using a dental microbrush and polymerized for 40 s with a broad-spectrum light-emitting diode (LED) curing unit (VALO Grand, Ultradent Products Inc., South Jordan, UT, USA) in manufacturer-specified standard power mode (nominal irradiance: 1000 mW/cm^2^), with the light tip positioned perpendicular and as close as possible to the surface [27,28,29].

#### 2.3.2. Mechanical Polishing

The mechanical polishing protocol was performed using the beige aluminum oxide-containing finishing spiral followed by the pink diamond-containing polishing spiral of the two-step Sof-Lex Diamond Finishing and Polishing System. No additional polishing paste was used. Both spirals were applied using a contra-angle handpiece connected to a micromotor (Marathon, Saeyang Microtech Co., Ltd., Daegu, Republic of Korea) at 20,000 rpm for 30 s per step under water cooling. A new set of finishing and polishing spirals was used for each experimental group. To minimize operator-related variability, all procedures were performed by the same operator using light, consistent manual pressure [30,31]. The spirals were kept in continuous motion over the entire experimental surface to maintain consistent surface contact. The specimens were subsequently ultrasonically cleaned in distilled water for 15 min.

Following the surface finishing protocols, the final thickness of each specimen was remeasured using a digital caliper, and all specimens remained within the predefined range of 1.50 ± 0.05 mm.

### 2.4. Baseline Color and Surface Roughness Measurements

#### 2.4.1. Color Measurement

Baseline color measurements were performed using a portable spectrophotometer (CM-2500d, Konica Minolta Sensing Inc., Sakai, Japan). Measurements were conducted in a dark environment within a color-measurement box lined with a neutral-gray material, with the specimens positioned on a neutral-gray background [32,33,34].

The spectrophotometer was set to the D65 standard illuminant, d/8° geometry, 10° standard observer, specular component excluded (SCE) mode, and an 8 mm measurement aperture [35]. The device was calibrated using the manufacturer’s calibration plate before each experimental group was measured. Three measurements were obtained from the same predetermined region centered on each specimen, and the mean CIELAB coordinates (L*, a*, and b*) were recorded as baseline values.

#### 2.4.2. Surface Roughness Measurement

Baseline mean surface roughness (Ra) was measured using a contact profilometer (MarSurf M 300 C and MarSurf RD 18 C; Mahr GmbH, Göttingen, Germany) equipped with a PHT 6-350/2 µm diamond stylus. Measurements were performed using a constant force of 0.7 mN, a traverse length of 1.75 mm, and a speed of 0.5 mm/s [14]. A cutoff length (λ_c_) of 0.25 mm with a Gaussian filter was used, and Ra was evaluated according to the International Organization for Standardization (ISO) 21920 standard [36]. The stylus movement was oriented perpendicular to the subsequent brushing direction.

The profilometer was calibrated before each group using a PRN-10 calibration standard (Mahr GmbH). Three measurements were initiated at positions 3, 6, and 9 mm along the 12 mm specimen edge, while maintaining the same measurement orientation, and their arithmetic mean was recorded as the baseline Ra value in µm.

### 2.5. Simulated Toothbrushing

After baseline measurements, the specimens were mounted in a multi-station brushing simulator (DentArge TB-6.1, Analitik Medikal, Gaziantep, Türkiye). Medium-bristle Powerdent Shine toothbrushes (İdeal Standart A.Ş., Istanbul, Türkiye) were positioned with the bristles contacting the experimental surfaces.

Colgate Total (Colgate-Palmolive Co., Guangzhou, China; reported RDA = 70) and Colgate Whitening (Colgate-Palmolive Co., Guangzhou, China; reported RDA = 124) were used as the two commercial toothpaste conditions [20]. Toothpaste slurries were prepared by mixing 6 g of toothpaste with 6 mL of distilled water at a 1:1 mass/volume ratio [37,38]. Each specimen was placed in a separate reservoir. Fresh slurry was prepared at room temperature for each specimen, mixed, and transferred to the corresponding reservoir. The slurry was neither renewed nor additionally mixed during the 10,000 brushing cycles. A new toothbrush was used for each specimen.

Brushing was performed at 25 °C under a constant 2.5 N load applied perpendicular to the specimen surface [39]. The simulator was set to perform linear reciprocating movements over a 10 mm path at 120 strokes/min (2 Hz). One brushing cycle was defined as one complete 10 mm forward and 10 mm backward movement [22]. Each specimen underwent 10,000 simulated toothbrushing cycles as a standardized laboratory exposure intended to approximate one year of toothbrushing [15,40,41]. Following toothbrushing, all specimens were rinsed with water, dried, and stored in a dry, dark environment at room temperature for 24 h before post-brushing measurements [42].

### 2.6. Post-Brushing Color and Surface Roughness Measurements

Color and surface roughness measurements were repeated after simulated toothbrushing using the same devices, calibration procedures, settings, measurement regions, orientations, and environmental conditions used at baseline.

### 2.7. Color Difference Calculation

Color differences between baseline and post-brushing measurements were calculated using the CIEDE2000 color-difference formula (Δ*E*_00_) [40,43]:

$$\Delta E_{00}={\left[{\left(\frac{\Delta L^{\prime }}{k_{L}S_{L}}\right)}^{2}+{\left(\frac{\Delta C^{\prime }}{k_{C}S_{C}}\right)}^{2}+{\left(\frac{\Delta H^{\prime }}{k_{H}S_{H}}\right)}^{2}+R_{T}\left(\frac{\Delta C^{\prime }}{k_{C}S_{C}}\right)\left(\frac{\Delta H^{\prime }}{k_{H}S_{H}}\right)\right]}^{1/2}$$

where Δ*L*′, Δ*C*′, and Δ*H*′ represent differences in lightness, chroma, and hue, respectively; *S_L_*, *S_C_*, and *S_H_* are weighting functions; and *R_T_* is the rotation term accounting for the interaction between chroma and hue. The parametric factors *k_L_*, *k_C_*, and *k_H_* were set to 1 [40,43,44].

For clinical interpretation, the 50:50% perceptibility and acceptability thresholds were Δ*E*_00_ = 0.8 and Δ*E*_00_ = 1.8, respectively [45]. Accordingly, Δ*E*_00_ < 0.8 was considered below the perceptibility threshold, 0.8 ≤ Δ*E*_00_ ≤ 1.8 perceptible but clinically acceptable, and Δ*E*_00_ > 1.8 above the clinical acceptability threshold.

### 2.8. Statistical Analysis

All 80 specimens were analyzed without exclusions using IBM SPSS Statistics 23.0, JASP 0.12, and R 4.2.1. Continuous variables are reported as mean ± standard deviation (SD). Assumptions were assessed using residual Shapiro–Wilk tests, quantile–quantile (Q–Q) plots, residual histograms, and median-centered Levene tests. Influential observations in the repeated-measures model were assessed; detailed diagnostics are provided in Supplementary File S1.

Δ*E*_00_ was analyzed using three-way factorial analysis of variance (ANOVA), with material–production system, surface finishing protocol, and toothpaste type as between-subject factors. Ra was analyzed using mixed repeated-measures ANOVA with the same factors and time (baseline/post-brushing) as the within-subject factor. All main effects and interactions were tested; no primary outcome or interaction was prespecified. Sphericity was not applicable because time had two levels.

Estimated marginal means were averaged across toothpaste types. Tukey adjustment covered six pairwise comparisons among the four material–production system × finishing combinations for Δ*E*_00_. Following the significant material–production system × finishing × time interaction for Ra, these combinations were compared at baseline, post-brushing, and for change (post-brushing minus baseline), with separate Tukey adjustments for each six-comparison family. Bonferroni adjustment covered the four within-combination time comparisons. Baseline L*, a*, and b* were compared across eight groups using separate one-way ANOVAs, with Holm adjustment across the three omnibus tests. Effect sizes were reported as partial eta squared (η_p_^2^), and contrasts as mean differences with 95% confidence intervals (CIs). Pairwise comparisons were two-sided; significance was set at *p* < 0.05.

## 3. Results

### 3.1. Color Difference Results

Baseline color coordinates measured after surface finishing and before brushing are presented in Supplementary Table S2. No overall group differences were detected after Holm adjustment for the three coordinates (adjusted *p* = 0.275, 0.465, and 0.099 for L*, a*, and b*, respectively). A total of 80 Δ*E*_00_ values, with 10 specimens from each of the eight experimental groups, were included in the color difference analysis. No observations were excluded from the analysis.

The residuals of the three-way factorial ANOVA model did not significantly deviate from a normal distribution (*W* = 0.993; *p* = 0.959). The normal Q–Q plot showed that the residuals were distributed around the reference line, and the residual histogram was approximately symmetric. The median-centered Levene test indicated no significant difference in variance among the groups, *F*(7, 72) = 1.974; *p* = 0.070. Although one observation was identified as a potential outlier based on the externally studentized residual, examination of the raw data confirmed that it represented a valid measurement; therefore, it was retained in the analysis.

**Figure 2:**
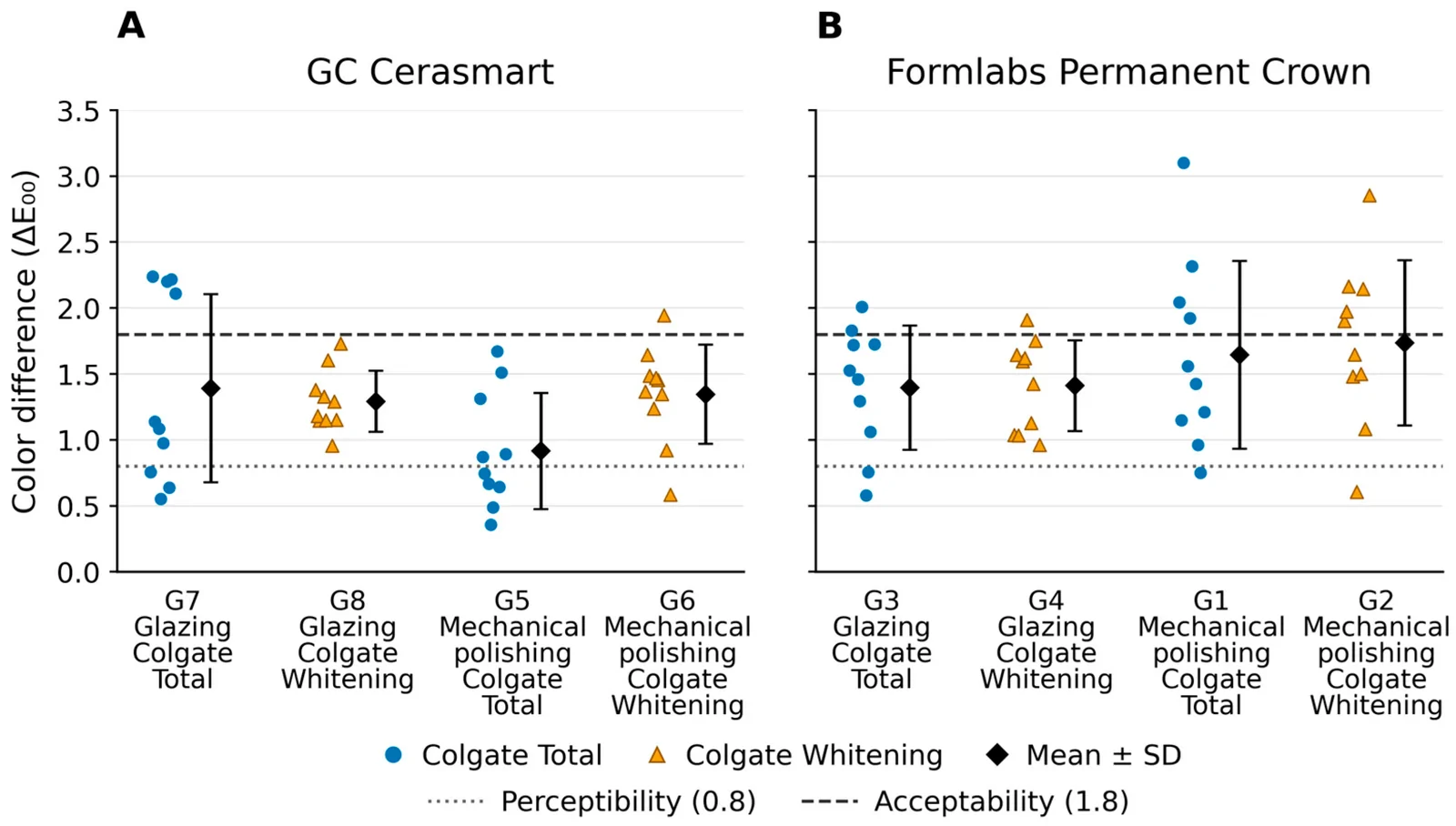
Individual Δ*E*_00_ values according to material–production system, surface finishing protocol, and toothpaste type. Panels (**A**) and (**B**) show GC Cerasmart and Formlabs Permanent Crown, respectively. Both panels use the same y-axis scale. Symbols represent individual specimens; black diamonds and error bars indicate mean ± standard deviation. Horizontal dotted and dashed lines indicate the perceptibility (0.8) and acceptability (1.8) thresholds, respectively. *n* = 10 per experimental group; total analysis population, *N* = 80.

Mean Δ*E*_00_ values ranged from 0.92 to 1.74 across the eight experimental groups (Table 1; Figure 2), and all group means were above the perceptibility threshold of 0.8 and below the acceptability threshold of 1.8. At the specimen level, 13 of 80 specimens (16.25%) were below the perceptibility threshold, 50 (62.50%) were between the perceptibility and acceptability thresholds, and 17 (21.25%) exceeded the acceptability threshold. Group-specific counts and percentages are presented in Supplementary Table S3.

The three-way factorial ANOVA revealed a significant material–production system × surface finishing protocol interaction for Δ*E*_00_ (Table 2), *F*(1,72) = 4.631, *p* = 0.035, partial η^2^ = 0.060, indicating that material-related differences depended on the finishing protocol. Accordingly, the significant main effect of material–production system, *F*(1,72) = 7.208, *p* = 0.009, partial η^2^ = 0.091, was interpreted in the context of this interaction. The main effects of surface finishing protocol (*F*(1,72) = 0.108, *p* = 0.744) and toothpaste type (*F*(1,72) = 0.895, *p* = 0.347) were not statistically significant, and the remaining interactions were also nonsignificant (*p* > 0.05). Estimated marginal means and 95% confidence intervals for the material–production system × surface finishing protocol interaction are presented in Table 3.

Tukey-adjusted simple-effect comparisons showed that, among mechanically polished specimens, the estimated marginal mean Δ*E*_00_ was significantly higher for Formlabs Permanent Crown than for GC Cerasmart (mean difference = 0.56; 95% CI: 0.13–0.99; *p* = 0.006). No significant between-material difference was observed for glazed specimens (mean difference = 0.06; 95% CI: −0.37–0.49; *p* = 0.982). Within GC Cerasmart, mechanical polishing did not differ significantly from glazing (mean difference = −0.21; 95% CI: −0.64–0.22; *p* = 0.572). Similarly, within Formlabs Permanent Crown, mechanical polishing did not differ significantly from glazing (mean difference = 0.29; 95% CI: −0.14–0.72; *p* = 0.304).

### 3.2. Surface Roughness Results

A total of 80 specimens, with 10 specimens from each of the eight experimental groups, were included in the surface roughness analysis. For each specimen, the mean of three profilometer measurements was used to calculate Ra values before brushing and after 10,000 brushing cycles. No specimens were excluded from the analysis.

The assumptions of the mixed-design repeated-measures ANOVA were evaluated using model residuals. Residuals for the subject means (W = 0.995; *p* = 0.985) and for the brushing-related change scores (W = 0.991; *p* = 0.832) did not significantly deviate from a normal distribution. The median-centered Levene test indicated that the assumption of homogeneity of variance was satisfied for both the subject means, *F*(7, 72) = 0.782; *p* = 0.605, and the change scores, *F*(7, 72) = 1.126; *p* = 0.356. Because the time factor consisted of only two measurement levels, assessment of the sphericity assumption was not required. One observation was flagged by the influence diagnostics; however, leave-one-out sensitivity analysis showed that the significant material–production system × surface finishing protocol × time interaction remained significant after exclusion of any individual specimen (*p* ≤ 0.0034).

**Figure 3:**
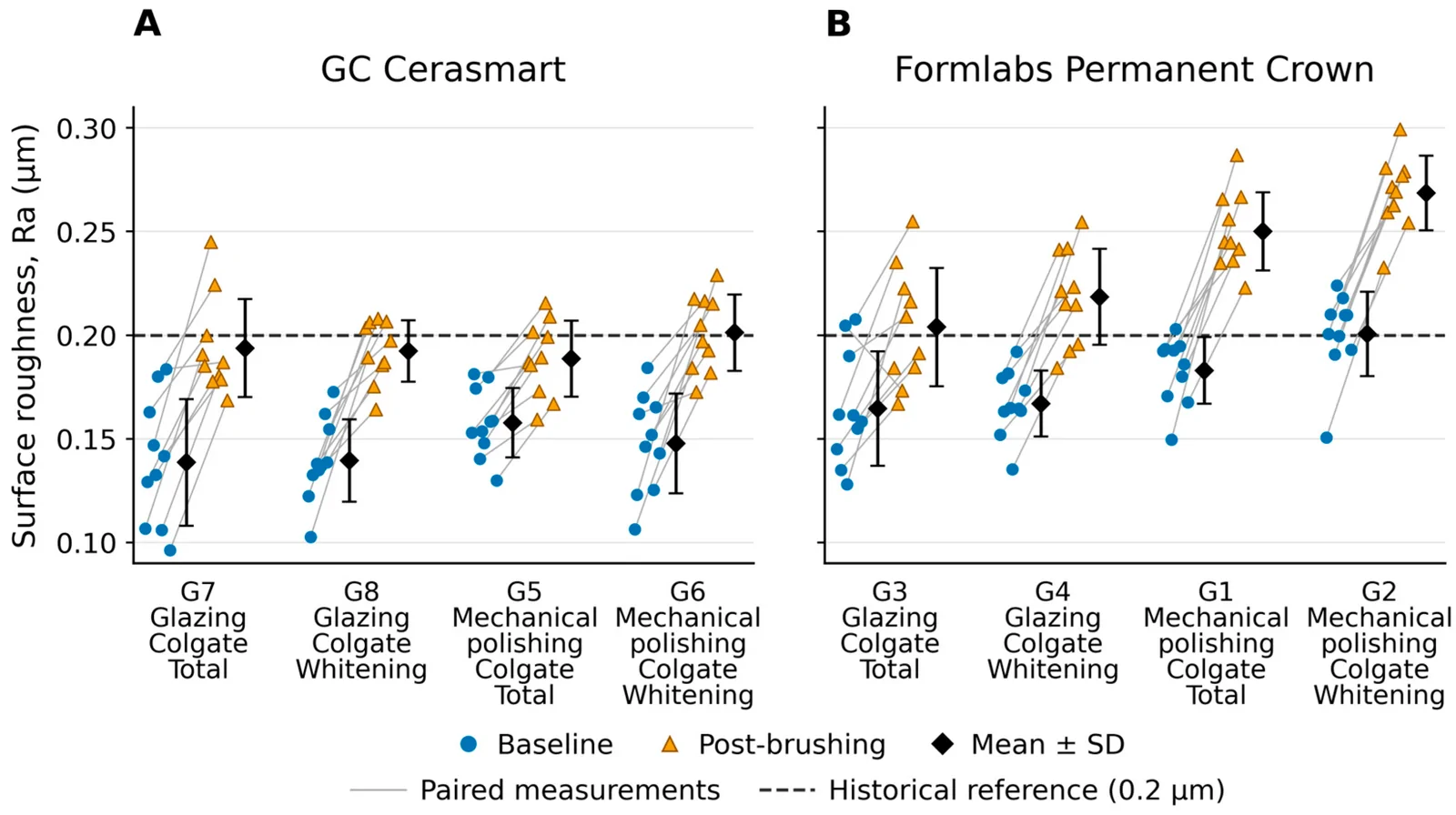
Individual pre- and post-brushing Ra values according to material–production system, surface finishing protocol, and toothpaste type. Panels (**A**) and (**B**) show GC Cerasmart and Formlabs Permanent Crown, respectively. Both panels use the same y-axis scale. Gray lines connect paired measurements from the same specimen; black diamonds and error bars indicate mean ± standard deviation. The horizontal dashed line indicates the historical 0.2 µm reference value. *n* = 10 per experimental group; total analysis population, *N* = 80.

Mean Ra values increased after toothbrushing in all eight experimental groups (Figure 3), with mean changes ranging from 0.031 to 0.068 µm; group-specific change scores and their 95% confidence intervals are presented in Table 4. At the specimen level, 9 of 80 specimens (11.25%) exceeded the historical 0.2 µm reference value at baseline, compared with 46 of 80 specimens (57.50%) after brushing. Group-specific counts and percentages are presented in Supplementary Table S4.

As shown in Table 5, four-factor mixed-design repeated-measures ANOVA revealed significant main effects of material–production system, *F*(1, 72) = 85.414, *p* < 0.001, partial η^2^ = 0.543; surface finishing protocol, *F*(1, 72) = 31.225, *p* < 0.001, partial η^2^ = 0.302; and brushing time, *F*(1, 72) = 395.277, *p* < 0.001, partial η^2^ = 0.846, on Ra values (Table 5). In contrast, the main effect of toothpaste type was not statistically significant, *F*(1, 72) = 2.951, *p* = 0.090.

A significant interaction was observed between the material–production system and surface finishing protocol, *F*(1, 72) = 13.306, *p* < 0.001, partial η^2^ = 0.156. In addition, the material–production system × surface finishing protocol × brushing time interaction was statistically significant, *F*(1, 72) = 10.434, *p* = 0.002, partial η^2^ = 0.127. This finding indicates that the brushing-related change in Ra depended on the combination of material–production system and surface finishing protocol. Therefore, the significant main effects were interpreted in the context of this three-way interaction. All remaining interactions were not statistically significant (*p* > 0.05).

Estimated marginal means and 95% confidence intervals for baseline Ra, post-brushing Ra, and their corresponding changes across material–production system and surface finishing protocol combinations are presented in Table 6.

Post hoc comparisons showed that post-brushing Ra values were significantly higher than pre-brushing values in all four material–surface finishing combinations (*p* < 0.001).

Before brushing, the Ra value of the glazed Formlabs Permanent Crown group was significantly higher than that of the glazed GC Cerasmart group (*p* = 0.001). Similarly, the mechanically polished Formlabs Permanent Crown group showed a significantly higher Ra value than the mechanically polished GC Cerasmart group (*p* < 0.001). No significant difference was observed between the glazing and mechanical polishing groups within GC Cerasmart (*p* = 0.212), whereas the mechanically polished Formlabs Permanent Crown group showed a significantly higher Ra value than the glazed Formlabs Permanent Crown group (*p* = 0.002).

After brushing, the Ra value of the glazed Formlabs Permanent Crown group remained significantly higher than that of the glazed GC Cerasmart group (*p* = 0.037). The mechanically polished Formlabs Permanent Crown group also showed a significantly higher Ra value than the mechanically polished GC Cerasmart group (*p* < 0.001). No significant difference was observed between glazing and mechanical polishing within GC Cerasmart (*p* = 0.992). In contrast, the post-brushing Ra value of the mechanically polished Formlabs Permanent Crown group was significantly higher than that of the glazed Formlabs Permanent Crown group (*p* < 0.001). Tukey-adjusted pairwise comparisons of the brushing-related change in Ra showed that mechanically polished Formlabs Permanent Crown exhibited a significantly greater increase than mechanically polished GC Cerasmart (mean difference = 0.025 µm; 95% CI: 0.006–0.045 µm; *p* = 0.006). Within Formlabs Permanent Crown, the increase was also significantly greater with mechanical polishing than with glazing (mean difference = 0.022 µm; 95% CI: 0.003–0.042 µm; *p* = 0.020). In contrast, the change did not differ significantly between the glazed Formlabs Permanent Crown and GC Cerasmart groups (mean difference = −0.009 µm; 95% CI: −0.028–0.011 µm; *p* = 0.652) or between mechanical polishing and glazing within GC Cerasmart (mean difference = −0.012 µm; 95% CI: −0.031–0.008 µm; *p* = 0.395).

## 4. Discussion

The present study evaluated the combined effects of surface finishing protocol and toothpaste type on color difference and surface roughness after 10,000 cycles of simulated toothbrushing in subtractively manufactured GC Cerasmart and additively manufactured Formlabs Permanent Crown material–production systems. Overall, the findings showed that the material–production system was significantly associated with both outcomes, whereas the effect of surface finishing depended on the material and the outcome evaluated. For Δ*E*_00_, the significant material–production system × surface finishing protocol interaction indicated that material-related differences depended on the finishing protocol; therefore, the material main effect was interpreted within the context of this interaction. Toothpaste type was not significant. For Ra, brushing time showed the largest effect, while the material–production system and surface finishing protocol also had significant effects. In addition, the significant material–production system × surface finishing protocol × brushing time interaction indicated that the surface response to brushing could not be generalized across finishing protocols. Accordingly, the null hypotheses were partially rejected.

The mean Δ*E*_00_ values of the eight experimental groups ranged from 0.92 to 1.74. According to the thresholds adopted in this study, all group means were perceptible but remained below the clinical acceptability threshold. Thus, 10,000 brushing cycles produced a measurable optical change, although the magnitude of this change was not clinically unacceptable under the tested conditions. However, this interpretation applies only to group means and does not imply clinical acceptability for every specimen. Specimen-level distributions relative to the perceptibility and acceptability thresholds are presented by group as counts and percentages in Supplementary Table S3. Previous studies have similarly shown that color stability after toothbrushing, thermal aging, and exposure to staining media depends not only on the manufacturing approach but also on the specific material formulation and surface treatment protocol [46,47,48].

The significant main effect of the material–production system on Δ*E*_00_ should be interpreted in the context of the significant interaction between material–production system and surface finishing protocol. Mechanically polished Formlabs Permanent Crown showed greater color change than mechanically polished GC Cerasmart (mean difference = 0.56; Tukey-adjusted 95% CI: 0.13–0.99), whereas no significant difference was observed between the glazed groups. This difference should be interpreted alongside the finding that all group means remained below the adopted clinical acceptability threshold. This pattern suggests that the optical response to brushing was dependent on the combination of material–production system and finishing protocol rather than on either factor alone. Because the within-material comparisons between glazing and mechanical polishing protocols were not significant for color difference, however, glazing protocol cannot be considered universally superior for maintaining color stability. Previous studies have likewise shown that the effects of glazing, coating, and polishing on color stability are material- and protocol-dependent [16], and similar material-specific behavior has been reported for roughness, gloss, and other surface properties [49,50,51].

The greater color difference observed in the mechanically polished Formlabs Permanent Crown group may be related to the combined influence of resin formulation, microstructure, printing conditions, post-polymerization procedures, and the finishing protocol. The optical behavior of 3D-printed resins has been reported to depend on material- and manufacturing-related variables [2], while microstructural differences between additively and subtractively manufactured restorative materials have also been demonstrated [5]. Post-polymerization conditions may influence color response [6], and polishing protocols may affect color stability after aging [48]. Nevertheless, these factors were not independently evaluated in the present study and should therefore be regarded as plausible mechanisms rather than direct causal explanations. Moreover, because each manufacturing approach was represented by only one commercial material, the effects of material composition and manufacturing method cannot be separated within the present design [2,5,48].

Neither the main effect of toothpaste type on Δ*E*_00_ nor any interaction involving toothpaste type was statistically significant. The two commercial toothpastes were selected to represent different reported RDA values; however, these nonsignificant findings should not be interpreted as evidence of equivalence between the two products or as evidence of an independent RDA effect. RDA is an abrasivity index defined for dentin, whereas the response of resin-based restorative materials may also be affected by the type, size, morphology, and concentration of abrasive particles, as well as by other chemical components, brushing load, and the matrix–filler structure of the restorative material. Current systematic and experimental evidence supports the view that the effects of dentifrice formulations on restorative materials cannot be predicted from RDA values alone [52,53].

A consistent finding for surface roughness was the significant increase in Ra after brushing across all four material–surface finishing combinations. The large effect associated with brushing time indicates that repeated toothbrushing was a major determinant of the observed surface changes. This finding is consistent with previous studies and systematic evidence showing that simulated toothbrushing can increase surface roughness in resin-containing CAD/CAM and 3D-printed restorative materials, with the magnitude of change varying according to material composition, brushing duration, and dentifrice characteristics [46,52,54].

Formlabs Permanent Crown exhibited higher Ra values than GC Cerasmart both before and after brushing; therefore, post-brushing differences cannot be attributed entirely to brushing. Mechanically polished Formlabs Permanent Crown nevertheless showed a greater brushing-related increase in Ra than mechanically polished GC Cerasmart (difference in increases = 0.025 µm; Tukey-adjusted 95% confidence interval (CI): 0.006–0.045 µm). This interval supports a greater increase under the tested conditions while indicating uncertainty about its magnitude; the clinical consequences of this difference were not evaluated. These findings are consistent with recent comparative data reporting greater surface roughness for Formlabs Permanent Crown [55]. Other studies have also demonstrated that surface characteristics after aging may vary according to the restorative material [56,57] and that polishing protocols can influence roughness and wettability [58]. Nevertheless, because the manufacturing method and commercial material were inherently linked in this study, the observed differences should not be attributed solely to additive manufacturing.

The Ra differences between the complete finishing protocols were material-specific: glazed Formlabs Permanent Crown specimens had lower Ra than mechanically polished specimens both before and after brushing, whereas no significant difference between finishing protocols was detected for GC Cerasmart at either time point. The lower Ra of glazed Formlabs Permanent Crown specimens may reflect an intact glaze coating rather than improved finishing of the underlying substrate. Because glaze thickness and coating loss were not measured, however, this finding should not be interpreted as direct evidence of a protective mechanism. Previous studies have reported that glazing or varnishing can reduce Ra in 3D-printed resins [16,49], although the magnitude of this effect depends on the material, application protocol, and aging conditions [16,50,51].

The historical Ra value of 0.2 µm has been discussed as a reference below which further reductions in surface roughness may not produce additional reductions in bacterial accumulation [59]. In the present study, this value was used only as a comparative reference rather than as an absolute biological threshold. After brushing, the pooled GC Cerasmart means for glazing (0.193 µm) and mechanical polishing (0.195 µm) remained below this reference value, whereas the Formlabs Permanent Crown means for glazing (0.211 µm) and particularly mechanical polishing (0.259 µm) exceeded it. Although these findings may be relevant when selecting and maintaining finishing protocols for the tested Formlabs Permanent Crown material–production system, they should not be interpreted as direct evidence of increased biological risk because biofilm formation and plaque retention were not measured in this study.

Neither the main effect of toothpaste type on Ra nor the toothpaste type × time interaction was statistically significant. However, these findings do not exclude smaller toothpaste-related effects and should not be interpreted as evidence of equivalence. Because only two commercial dentifrices were evaluated, the effects of RDA cannot be separated from those of the remaining formulation components. Therefore, clinical recommendations should not be based solely on the RDA classification of a dentifrice but should also consider the restorative material and the overall dentifrice formulation [52,53].

The main limitation of this in vitro study is the use of flat specimens, which do not fully reproduce clinical crown geometry or the complex interaction of toothbrush bristles with marginal and occlusal surfaces. Salivary pellicle, fluctuations in temperature and pH, staining beverages, masticatory loading, and concurrent hydrothermal aging were not included in the experimental model. Accordingly, the clinical implications of the observed surface roughness differences for material-specific finishing selection, monitoring of restoration surfaces, and repolishing or surface renewal should be interpreted cautiously.

The use of only two materials, two surface finishing protocols, and two dentifrices limits external validity. Translucency was not measured; therefore, material-dependent differences in translucency may have influenced the measured color coordinates. The absence of an a priori sample-size calculation based on a prespecified primary outcome and clinically relevant effect should be considered a limitation; moreover, with 10 specimens per experimental cell, the statistical power for higher-order interactions may have been limited. In addition, manufacturing method was confounded with the commercial material; consequently, the independent effects of additive and subtractive manufacturing cannot be determined. Potential dependence among specimens originating from the same milling block or print run was not accounted for in the analysis. Because only a 90° build orientation and a 50 µm layer thickness were evaluated, the findings may not be generalizable to other clinically used printing orientations or layer thicknesses. Surface characterization was limited to contact profilometry and Ra measurements, whereas three-dimensional topographic parameters, gloss, volumetric wear, glaze thickness, surface morphology, and biofilm formation were not evaluated. Intra-operator repeatability and measurement error were not independently quantified for the color and surface roughness measurements, which limits the assessment of measurement precision. Finally, although 10,000 brushing cycles provided a standardized basis for comparison, this number should not be considered a direct one-to-one representation of clinical time.

Future studies should include multiple materials within the same manufacturing approach and different manufacturing systems within the same material class to better distinguish material-related effects from manufacturing-related effects. Printing orientation, layer thickness, washing procedures, and post-polymerization conditions should be evaluated as independent factors, and long-term toothbrushing should be combined with thermal, chemical, and mechanical aging. Experimental dentifrices formulated with the same abrasive system but different RDA levels may also help isolate the independent effect of RDA from the effects of other formulation components.

## 5. Conclusions

Under the tested in vitro conditions, the findings were specific to the GC Cerasmart and Formlabs Permanent Crown material–production systems, the glazing and mechanical polishing protocols, and the two commercial toothpastes evaluated. Mean Δ*E*_00_ values remained below the acceptability threshold in all groups, although some individual specimens exceeded it. Mechanically polished Formlabs Permanent Crown showed greater color change than mechanically polished GC Cerasmart. Ra increased after brushing across all material–finishing combinations. Glazed Formlabs Permanent Crown showed lower Ra than mechanically polished specimens, whereas no significant post-brushing difference between finishing protocols was detected for GC Cerasmart. No statistically significant difference between the two toothpaste conditions was detected for either outcome; this does not establish equivalence. Long-term in vivo studies are needed to confirm the clinical applicability of these findings.

## Figures and Tables

**Table 1:** Δ*E*_00_ values according to material–production system, surface finishing protocol, and toothpaste type.

Material–Production System	Surface Finishing Protocol	Colgate Total, Mean ± SD (95% CI)	Colgate Whitening, Mean ± SD (95% CI)
GC Cerasmart	Glazing	1.39 ± 0.71 (0.88–1.90)	1.29 ± 0.23 (1.13–1.46)
GC Cerasmart	Mechanical polishing	0.92 ± 0.44 (0.60–1.23)	1.35 ± 0.38 (1.08–1.61)
Formlabs Permanent Crown	Glazing	1.40 ± 0.47 (1.06–1.73)	1.41 ± 0.34 (1.16–1.66)
Formlabs Permanent Crown	Mechanical polishing	1.64 ± 0.71 (1.13–2.15)	1.74 ± 0.63 (1.29–2.18)

Values are presented as mean ± standard deviation, with 95% confidence intervals in parentheses. The 95% confidence intervals are pointwise Student’s *t* intervals for the group means (df = 9). *n* = 10 per experimental group; total analysis population, *N* = 80.

**Table 2:** Three-way factorial ANOVA results for Δ*E*_00_ values.

Source of Variation	df	SS	MS	F	*p*	Partial η^2^
Material–production system	1	1.924	1.924	7.208	**0.009**	0.091
Surface finishing protocol	1	0.029	0.029	0.108	0.744	0.001
Toothpaste type	1	0.239	0.239	0.895	0.347	0.012
Material–production system × surface finishing protocol	1	1.236	1.236	4.631	**0.035**	0.060
Material–production system × Toothpaste type	1	0.063	0.063	0.236	0.629	0.003
Surface finishing protocol × Toothpaste type	1	0.459	0.459	1.719	0.194	0.023
Material–production system × surface finishing protocol × Toothpaste type	1	0.256	0.256	0.958	0.331	0.013
Error	72	19.221	0.267	—	—	—
Corrected total	79	23.427	—	—	—	—

SS: sum of squares; MS: mean square; df: degrees of freedom. Bold *p* values indicate statistical significance. Total analysis population, *N* = 80.

**Table 3:** Estimated marginal means and 95% confidence intervals for the material–production system × surface finishing protocol interaction for Δ*E*_00_.

Material–Production System	Surface Finishing Protocol	Estimated Marginal Mean	95% CI	Tukey Group
GC Cerasmart	Mechanical polishing	1.13	0.90–1.36	b
GC Cerasmart	Glazing	1.34	1.11–1.57	ab
Formlabs Permanent Crown	Mechanical polishing	1.69	1.46–1.92	a
Formlabs Permanent Crown	Glazing	1.40	1.17–1.63	ab

Estimated marginal means with 95% confidence intervals are presented, averaged across toothpaste types. Each material–production system × surface finishing protocol combination includes 20 specimens (*n* = 20); total analysis population, *N* = 80. Groups that do not share the same letter differ significantly in pairwise comparisons adjusted using Tukey’s honestly significant difference (HSD) method. CI: confidence interval.

**Table 4:** Pre-brushing, post-brushing, and change in Ra values according to material–production system, surface finishing protocol, and toothpaste type.

Material–Production System	Surface Finishing Protocol	Toothpaste Type	Pre-Brushing Ra, Mean ± SD (95% CI), µm	Post-Brushing Ra, Mean ± SD (95% CI), µm	Change in Ra, Mean (95% CI), µm
GC Cerasmart	Glazing	Colgate Total	0.139 ± 0.031 (0.117–0.161)	0.194 ± 0.024 (0.177–0.211)	0.055 (0.035–0.075)
GC Cerasmart	Glazing	Colgate Whitening	0.140 ± 0.020 (0.125–0.154)	0.192 ± 0.015 (0.182–0.203)	0.053 (0.036–0.070)
GC Cerasmart	Mechanical polishing	Colgate Total	0.158 ± 0.017 (0.146–0.170)	0.189 ± 0.018 (0.176–0.202)	0.031 (0.021–0.041)
GC Cerasmart	Mechanical polishing	Colgate Whitening	0.148 ± 0.024 (0.131–0.165)	0.201 ± 0.018 (0.188–0.214)	0.053 (0.035–0.072)
Formlabs Permanent Crown	Glazing	Colgate Total	0.165 ± 0.028 (0.145–0.184)	0.204 ± 0.029 (0.183–0.224)	0.039 (0.015–0.063)
Formlabs Permanent Crown	Glazing	Colgate Whitening	0.167 ± 0.016 (0.156–0.178)	0.218 ± 0.023 (0.202–0.235)	0.051 (0.038–0.065)
Formlabs Permanent Crown	Mechanical polishing	Colgate Total	0.183 ± 0.016 (0.171–0.195)	0.250 ± 0.019 (0.237–0.264)	0.067 (0.051–0.083)
Formlabs Permanent Crown	Mechanical polishing	Colgate Whitening	0.201 ± 0.020 (0.186–0.215)	0.269 ± 0.018 (0.256–0.281)	0.068 (0.057–0.079)

Pre- and post-brushing values are presented as mean ± standard deviation, with 95% confidence intervals in parentheses. The corresponding 95% confidence intervals are pointwise Student’s *t* intervals for the group means (df = 9). Change scores are presented as mean change (post-brushing minus baseline) with 95% confidence intervals based on paired measurements. Ra: arithmetic mean surface roughness. *n* = 10 per experimental group; total analysis population, *N* = 80.

**Table 5:** Four-factor mixed-design repeated-measures ANOVA results for Ra values.

Source of Variation	df	F	*p*	Partial η^2^
Material–production system	1	85.414	**<0.001**	0.543
Surface finishing protocol	1	31.225	**<0.001**	0.302
Toothpaste type	1	2.951	0.090	0.039
Brushing time	1	395.277	**<0.001**	0.846
Material–production system × brushing time	1	2.512	0.117	0.034
Surface finishing protocol × brushing time	1	0.984	0.324	0.013
Toothpaste type × brushing time	1	2.512	0.117	0.034
Material–production system × surface finishing protocol	1	13.306	**<0.001**	0.156
Material–production system × Toothpaste type	1	2.525	0.116	0.034
Surface finishing protocol × Toothpaste type	1	0.481	0.490	0.007
Material–production system × surface finishing protocol × brushing time	1	10.434	**0.002**	0.127
Material–production system × Toothpaste type × brushing time	1	0.113	0.738	0.002
Surface finishing protocol × Toothpaste type × brushing time	1	0.414	0.522	0.006
Material–production system × surface finishing protocol × Toothpaste type	1	0.253	0.617	0.003
Material–production system × surface finishing protocol × Toothpaste type × brushing time	1	2.941	0.091	0.039

df: numerator degrees of freedom; denominator degrees of freedom = 72 for all F tests. Bold *p* values indicate statistical significance. Total analysis population, *N* = 80.

**Table 6:** Estimated marginal means and 95% confidence intervals for Ra before and after toothbrushing and for the corresponding changes.

Material–Production System	Surface Finishing Protocol	Baseline Ra, µm (95% CI)	Post-Brushing Ra, µm (95% CI)	Change in Ra, µm (95% CI)
GC Cerasmart	Mechanical polishing	0.153 (0.143–0.163)	0.195 (0.186–0.204)	0.042 (0.032–0.053)
GC Cerasmart	Glazing	0.139 (0.129–0.149)	0.193 (0.184–0.202)	0.054 (0.044–0.064)
Formlabs Permanent Crown	Mechanical polishing	0.192 (0.182–0.202)	0.259 (0.250–0.269)	0.068 (0.057–0.078)
Formlabs Permanent Crown	Glazing	0.166 (0.156–0.176)	0.211 (0.202–0.220)	0.045 (0.035–0.056)

Values are model-based estimated marginal means with 95% confidence intervals, averaged across toothpaste types. Change was calculated as post-brushing minus baseline Ra, accounting for paired measurements. CI: confidence interval. Each material–production system × surface finishing protocol combination includes 20 specimens (*n* = 20); total analysis population, *N* = 80. Between-combination pairwise comparisons at baseline, post-brushing, and for change scores were Tukey-adjusted separately within each family of six comparisons. Within-combination baseline-to-post-brushing comparisons were Bonferroni-adjusted across the four comparisons.

## Data Availability

The raw data supporting the conclusions of this article will be made available by the authors on request.

## Supplementary Materials

The following supporting information can be downloaded at https://www.mdpi.com/article/10.3390/dj14100674/s1. Table S1: Materials and products used in the study, including manufacturers, lot numbers, compositions, filler/abrasive contents, and relevant processing recommendations; Table S2: Baseline characteristics of the experimental groups; Table S3: Distribution of specimen-level ΔE_00_ values according to perceptibility and acceptability thresholds by experimental group; Table S4: Distribution of specimens exceeding the historical Ra reference value of 0.2 µm at baseline and after brushing; Figure S1: Preparation of GC Cerasmart 270 specimens: (1) precision cutting, (2) grinding, and (3) ultrasonic cleaning; Figure S2: Preparation of Formlabs Permanent Crown specimens: (1) 3D printing, (2) washing, and (3) first post-curing; File S1: Full Statistical Model Output for Surface Roughness (Ra); File S2: Specimen-level color and surface roughness data, group assignments, and reproducible R analysis code.

## Abbreviations

The following abbreviations are used in this manuscript:

ANOVA	Analysis of variance
CAD/CAM	Computer-aided design/computer-aided manufacturing
HSD	Honestly significant difference
LFS	Low Force Stereolithography
RDA	Relative dentin abrasivity
SCE	Specular component excluded
Δ*E*_00_	CIEDE2000 color difference
Ra	Arithmetic mean surface roughness
3D	Three-dimensional
CI	Confidence interval
CIE	International Commission on Illumination
CS	GC Cerasmart 270
df	Degrees of freedom
FL	Formlabs Permanent Crown
ISO	International Organization for Standardization
LED	Light-emitting diode
MS	Mean square
Q-Q	Quantile–quantile
SD	Standard deviation
SS	Sum of squares
