# Supplementary material for: Color Stability and Surface Roughness of Two Restorative Material–Production Systems: Effects of Surface Finishing and Brushing with Two Commercial Toothpastes with Different RDA Classifications
Source: Dent J (Basel). 2026 Oct 16;14(10):674. doi: 10.3390/dj14100674 (PMC13702949; doi:10.3390/dj14100674)

Supplementary Table S1. Materials and products used in the study, including manufacturers, lot numbers, compositions, filler/abrasive contents, and relevant processing recommendations

Product	Product category	Manufacturer	Lot number	Composition/filler information	Manufacturer's relevant processing recommendations
GC Cerasmart 270	Milled resin-composite block	GC Corporation, Tokyo, Japan	2405206	Bis-MEPP, UDMA, and DMA with 71 wt% silica (20 nm) and barium glass (300 nm) nanoparticles	Mill using a composite resin block program; finish and polish with coarse and fine silicone points. For glazing, air-abrade with 25–50 µm alumina at 1.5 bar, clean and dry, and apply a primer before OPTIGLAZE Color.
Formlabs Permanent Crown	3D-printed definitive restorative resin	Formlabs Inc., Somerville, MA, USA	600980	Ethoxylated bisphenol A dimethacrylate (Bis-EMA)-based resin matrix; silanized dental glass fillers, 30–50 wt%; diphenyl(2,4,6-trimethylbenzoyl)phosphine oxide (TPO) photoinitiator	Use material-specific printing settings followed by isopropyl alcohol washing and post-curing according to the manufacturer's validated workflow.
G-Multi Primer	Universal restorative primer	GC Corporation, Tokyo, Japan	2502074	Ethanol, dimethacrylate, 10-methacryloyloxydecyl dihydrogen phosphate (MDP), silane, methacryloyloxydecyl dihydrogen thiophosphate (MDTP)	Clean the prepared surface using an ultrasonic bath or alcohol, rinse, and dry with oil-free air. Then apply a thin layer of G-Multi Primer with a microbrush and air-dry.
OPTIGLAZE Color Clear	Light-polymerized surface coating	GC Corporation, Tokyo, Japan	2507071	Methyl methacrylate (30–40%), multifunctional acrylate (50–60%), silica filler (10%), diphenyl(2,4,6-trimethylbenzoyl)phosphine oxide (<5%), photoinitiator	Apply a thin, uniform layer with a brush and light-cure with a suitable curing unit. Do not air-

					blow after application.
Sof-Lex Diamond Polishing System	Two-step mechanical polishing system	3M, St. Paul, MN, USA	9656724	Thermoplastic elastomer spirals impregnated with aluminum oxide (pre-polishing) or diamond particles (final polishing)	Use the beige pre-polishing spiral first, followed by the pink diamond polishing spiral; recommended speed: 15,000–20,000 rpm; no polishing paste is required.
Colgate Total	Commercial dentifrice; lower-RDA condition	Colgate-Palmolive Co., Guangzhou, China	1348CH12J1	Hydrated silica, PVM/MA copolymer, sodium lauryl sulfate, water, glycerin, sorbitol, propylene glycol, cellulose gum, carrageenan, sodium hydroxide, sodium saccharin, flavor, titanium dioxide; 1450 ppm F. Abrasive system: hydrated silica	-
Colgate Whitening	Commercial dentifrice; higher-RDA condition	Colgate-Palmolive Co., Guangzhou, China	3312CN12L3	Calcium carbonate, hydrated silica, sodium monofluorophosphate, sodium bicarbonate, sodium carbonate, sodium lauryl sulfate, water, sorbitol, cellulose gum, magnesium aluminium silicate, benzyl alcohol, sodium saccharin, flavor, eugenol, limonene; 1450 ppm F. Abrasive system: calcium carbonate and hydrated silica	-
Powerdent Shine	Manual toothbrush	İdeal Standart A.Ş., Istanbul, Türkiye	-	Medium bristle	-

Abbreviations: Bis-EMA, ethoxylated bisphenol A dimethacrylate; Bis-MEPP, 2,2-bis(4-methacryloxypolyethoxyphenyl) propane; DMA, dimethacrylate; MDP, 10-methacryloyloxydecyl dihydrogen phosphate; MDTP, methacryloyloxydecyl dihydrogen thiophosphate; PVM/MA, polyvinyl methyl ether/maleic acid; TPO, diphenyl(2,4,6-trimethylbenzoyl)phosphine oxide; UDMA, urethane dimethacrylate; RDA, relative dentin abrasivity

Supplementary Table S2. Baseline characteristics of the experimental groups

Group	*n*	Baseline *L**, Mean ± SD	Baseline *a**, Mean ± SD	Baseline *b**, Mean ± SD	Baseline Ra (μm), Mean ± SD
G1	10	77.599 ± 1.188	−0.026 ± 0.392	11.781 ± 1.123	0.183 ± 0.016
G2	10	77.150 ± 0.726	−0.100 ± 0.402	12.000 ± 0.862	0.201 ± 0.020
G3	10	77.408 ± 0.859	−0.141 ± 0.253	12.313 ± 0.805	0.165 ± 0.028
G4	10	76.989 ± 0.716	−0.057 ± 0.262	12.107 ± 0.835	0.167 ± 0.016
G5	10	77.270 ± 0.891	−0.066 ± 0.438	11.471 ± 0.947	0.158 ± 0.017
G6	10	78.056 ± 1.018	0.223 ± 0.302	12.231 ± 0.860	0.148 ± 0.024
G7	10	77.779 ± 1.255	0.074 ± 0.371	11.743 ± 0.645	0.139 ± 0.031
G8	10	77.953 ± 0.811	−0.067 ± 0.504	12.784 ± 0.460	0.140 ± 0.020

Note: Values are presented as mean ± standard deviation (n = 10 per group; total analysis population, N = 80). Measurements were obtained after surface finishing and before brushing. Each specimen's Ra value represents the mean of three measurements. Group definitions for Supplementary Tables S2 and S3: G1–G4, Formlabs Permanent Crown; G5–G8, GC Cerasmart 270. G1, G2, G5, and G6 received mechanical polishing; G3, G4, G7, and G8 received glazing. Odd-numbered groups were assigned to Colgate Total, and even-numbered groups to Colgate Whitening.

Supplementary Table S3. Distribution of specimen-level ΔE_{00} values according to perceptibility and acceptability thresholds by experimental group.

**Group**	***n***	**ΔE_{00} < 0.8, *n* (%)**	**0.8 ≤ ΔE_{00} ≤ 1.8, *n* (%)**	**ΔE_{00} > 1.8, *n* (%)**
G1	10	1 (10%)	5 (50%)	4 (40%)
G2	10	1 (10%)	4 (40%)	5 (50%)
G3	10	2 (20%)	6 (60%)	2 (20%)
G4	10	0 (0%)	9 (90%)	1 (10%)
G5	10	5 (50%)	5 (50%)	0 (0%)
G6	10	1 (10%)	8 (80%)	1 (10%)
G7	10	3 (30%)	3 (30%)	4 (40%)
G8	10	0 (0%)	10 (100%)	0 (0%)
Total	80	13 (16.25%)	50 (62.50%)	17 (21.25%)

Values are presented as *n* (%). *n* = 10 per experimental group; total analysis population, *N* = 80.

Supplementary Table S4. Distribution of specimens exceeding the historical Ra reference value of 0.2 μm at baseline and after brushing.

Experimental group	*n*	Baseline > 0.2 μm, *n* (%)	Post-brushing > 0.2 μm, *n* (%)
FL–Mech–Colgate Total	10	1 (10%)	10 (100%)
FL–Mech– Colgate Whitening	10	6 (60%)	10 (100%)
FL–Glaze– Colgate Total	10	2 (20%)	5 (50%)
FL–Glaze– Colgate Whitening	10	0 (0%)	7 (70%)
CS–Mech– Colgate Total	10	0 (0%)	3 (30%)
CS–Mech– Colgate Whitening	10	0 (0%)	5 (50%)
CS–Glaze– Colgate Total	10	0 (0%)	2 (20%)
CS–Glaze– Colgate Whitening	10	0 (0%)	4 (40%)
Total	80	9 (11.25%)	46 (57.50%)

Abbreviations: FL, Formlabs Permanent Crown; CS, GC Cerasmart 270; Mech, mechanical polishing; Glaze, glazing; Ra, arithmetic mean surface roughness. The 0.2 μm value is presented as a historical comparative reference and should not be interpreted as a universal biological threshold. Biofilm accumulation was not measured in this study. Values equal to 0.2 μm were not classified as exceeding the reference value.

Figure S1. Preparation of GC Cerasmart 270 specimens: (1) precision cutting, (2) grinding, and (3) ultrasonic cleaning

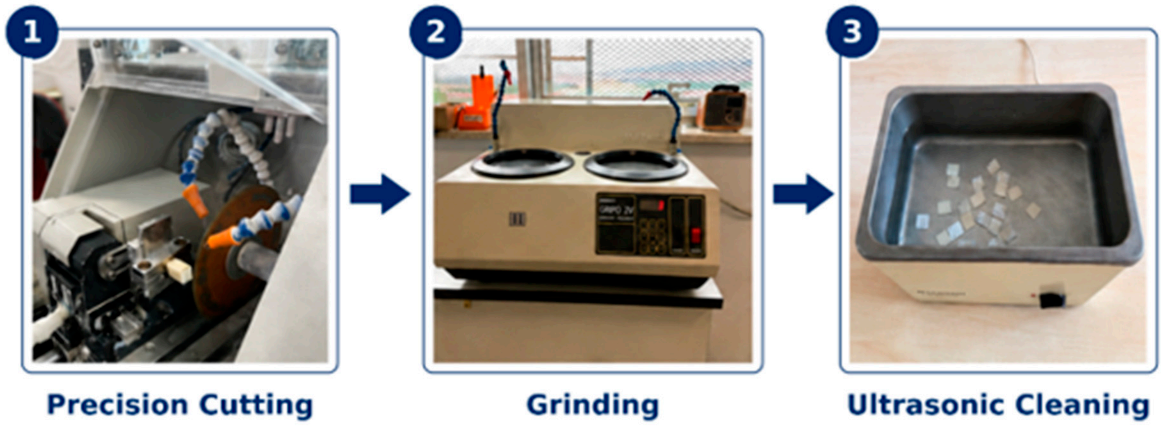

Figure S2. Preparation of Formlabs Permanent Crown specimens: (1) 3D printing, (2) washing, and (3) first post-curing.

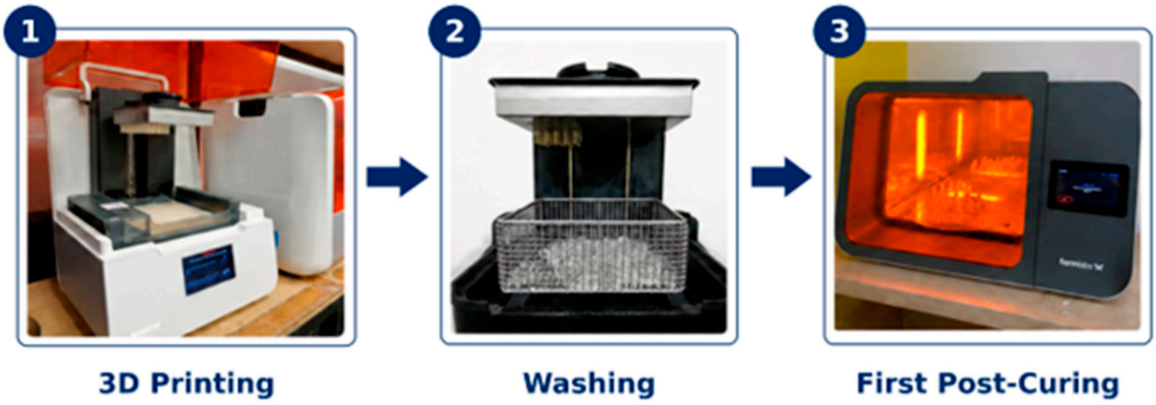

Supplement: Supplementary file 1 [file dentistry-14-00674-s001.zip › suplementary tables-figures..pdf]
