# Supplementary material for: Color Stability and Surface Roughness of Two Restorative Material–Production Systems: Effects of Surface Finishing and Brushing with Two Commercial Toothpastes with Different RDA Classifications
Source: Dent J (Basel). 2026 Oct 16;14(10):674. doi: 10.3390/dj14100674 (PMC13702949; doi:10.3390/dj14100674)
Supplement: Supplementary file 1 [file dentistry-14-00674-s001.zip › Supplementary File S1.pdf]

Supplementary File S1
Full Statistical Model Output for Surface Roughness (Ra)

Simulated toothbrushing study; N = 80 specimens

1. Model specification

Ra was analyzed using a mixed repeated-measures design with material–production system, surface finishing protocol, and toothpaste type as between-subject factors and time (baseline and post-brushing) as the within-subject factor. With two time points, the within-subject tests are equivalently represented by the brushing-related change (post-brushing minus baseline), while between-subject effects are represented by the subject mean across the two time points. Type III tests with sum/effect coding were used to reproduce the reported factorial repeated-measures ANOVA.

Factor levels: Material = Formlabs Permanent Crown / GC Cerasmart; surface finishing protocol = mechanical polishing / glazing; toothpaste type = Colgate Total / Colgate Whitening.

2. Between-subject effects

Effect	df	*F*	*p*	Partial η_p^2
Material–production system	1, 72	85.414	<0.001	0.543
Surface finishing protocol	1, 72	31.225	<0.001	0.302
Toothpaste type	1, 72	2.951	0.090	0.039
Material × finishing	1, 72	13.306	<0.001	0.156
Material × toothpaste	1, 72	2.525	0.116	0.034
Finishing × toothpaste	1, 72	0.481	0.490	0.007
Material × finishing × toothpaste	1, 72	0.253	0.617	0.003

3. Within-subject effects involving time

Effect	df	*F*	*p*	Partial η_p^2
Time	1, 72	395.277	<0.001	0.846
Material × time	1, 72	2.512	0.117	0.034
Finishing × time	1, 72	0.984	0.324	0.013
Toothpaste × time	1, 72	2.512	0.117	0.034
Material × finishing × time	1, 72	10.434	0.002	0.127
Material × toothpaste × time	1, 72	0.113	0.738	0.002
Finishing × toothpaste × time	1, 72	0.414	0.522	0.006
Material × finishing × toothpaste × time	1, 72	2.941	0.091	0.039

4. Model-based descriptive estimates

Material–production system	Surface finishing protocol	Baseline Ra (µm)	Post-brushing Ra (µm)	Change (µm)
Formlabs Permanent Crown	Glazing	0.166	0.211	0.045
Formlabs Permanent Crown	Mechanical polishing	0.192	0.259	0.068
GC Cerasmart	Glazing	0.139	0.193	0.054
GC Cerasmart	Mechanical polishing	0.153	0.195	0.042

Values are averaged across toothpaste types.

5. Tukey-adjusted between-combination contrasts

At baseline, after brushing, and for the change in Ra, the four material–production system × surface finishing protocol combinations were compared as separate families of six pairwise comparisons using Tukey adjustment.

Baseline Ra

Comparison	Mean difference	95% CI	Adjusted *p*	Significant
CS Mechanical − CS Glazing	0.0137	-0.0047 to 0.0320	0.212	No
FL Glazing − CS Glazing	0.0268	0.0084 to 0.0451	0.001	Yes
FL Mechanical − CS Glazing	0.0527	0.0344 to 0.0710	<0.001	Yes

FL Glazing − CS Mechanical	0.0131	-0.0052 to 0.0314	0.246	No
FL Mechanical − CS Mechanical	0.0391	0.0207 to 0.0574	<0.001	Yes
FL Mechanical − FL Glazing	0.0260	0.0076 to 0.0443	0.002	Yes

Post-brushing Ra

Comparison	Mean difference	95% CI	Adjusted *p*	Significant
CS Mechanical − CS Glazing	0.0019	-0.0154 to 0.0192	0.992	No
FL Glazing − CS Glazing	0.0181	0.0008 to 0.0355	0.037	Yes
FL Mechanical − CS Glazing	0.0663	0.0489 to 0.0836	<0.001	Yes
FL Glazing − CS Mechanical	0.0162	-0.0011 to 0.0336	0.075	No
FL Mechanical − CS Mechanical	0.0644	0.0470 to 0.0817	<0.001	Yes
FL Mechanical − FL Glazing	0.0482	0.0308 to 0.0655	<0.001	Yes

Change in Ra

Comparison	Mean difference	95% CI	Adjusted *p*	Significant
CS Mechanical − CS Glazing	-0.0118	-0.0313 to 0.0078	0.3950	No
FL Glazing − CS Glazing	-0.0087	-0.0282 to 0.0109	0.6517	No
FL Mechanical − CS Glazing	0.0136	-0.0060 to 0.0331	0.2714	No
FL Glazing − CS Mechanical	0.0031	-0.0164 to 0.0227	0.9750	No
FL Mechanical − CS Mechanical	0.0253	0.0058 to 0.0449	0.0059	Yes
FL Mechanical − FL Glazing	0.0222	0.0026 to 0.0418	0.0198	Yes

6. Bonferroni-adjusted within-combination baseline-to-post contrasts

Combination	Baseline	Post	Change	Contrast *t*	Bonferroni-adjusted *p*
CS – Glazing	0.139	0.193	0.054	10.267	<0.001
CS – Mechanical	0.153	0.195	0.042	8.029	<0.001
FL – Glazing	0.166	0.211	0.045	8.622	<0.001
FL – Mechanical	0.192	0.259	0.068	12.844	<0.001

All four baseline-to-post-brushing comparisons remained significant after Bonferroni adjustment (adjusted $p < 0.001$).

7. Influence diagnostics and sensitivity analysis

Influential observations for the brushing-related repeated-measures component were assessed using externally studentized residuals and Cook's distance. Leave-one-out sensitivity analysis was performed by refitting the factorial change model after excluding each specimen in turn.

Flagged observation	Material	Finishing	Toothpaste	Change	Externally studentized residual	Cook's distance
G3, specimen 3	Formlabs Permanent Crown	Glazing	Colgate Total	-0.0313	-3.385	0.139

One observation was flagged by the influence diagnostics. When this observation was excluded, the material–production system × surface finishing protocol × time interaction remained significant ($F = 9.222$, $p = 0.0033$). Across all 80 leave-one-out refits, the interaction F statistic ranged from 9.222 to 13.204, and the corresponding p values ranged from 0.0005 to 0.0033. Thus, the significant three-way interaction was not dependent on any single specimen.

8. Reproducibility note

The output above was generated from the specimen-level Ra data used for the manuscript. The material–production system × surface finishing protocol × time result ($F(1,72) = 10.434$, $p = 0.002$) reproduces the corresponding result reported in the manuscript. Values may differ in the final displayed decimal place because of rounding.
